# A case–control study of endogenous hormones and cervical cancer

**DOI:** 10.1038/sj.bjc.6601514

**Published:** 2004-01-06

**Authors:** T S Shields, R T Falk, R Herrero, M Schiffman, N S Weiss, C Bratti, A C Rodriguez, M E Sherman, R D Burk, A Hildesheim

**Affiliations:** 1 Hormonal and Reproductive Epidemiology Branch, Division of Cancer Epidemiology and Genetics, National Cancer Institute, NIH, DHHS, 6120 Executive Blvd, MSC 7234, Rockville, MD 20852, USA; 2 Proyecto Epidemiologico Guanacaste, Fundacion Costarricense para la Docencia en Ciencias del la Salud, PO Box 125-6151, San Jose, Costa Rica; 3 Department of Epidemiology, School of Public Health and Community Medicine, University of Washington, Box 357236 Seattle, WA 98195, USA; 4 Fred Hutchinson Cancer Research Center, 1100 Fairview Avenue North, Seattle, WA 98109, USA; 5 Department of Pediatrics, Microbiology and Immunology, Epidemiology and Population Health, and Obstetrics, Gynecology and Women's Health, Albert Einstein College of Medicine, 1300 Morris Park Avenue, Bronx, NY 10461-1602, USA

**Keywords:** DHEAS, oestradiol, oestrone, oestrone sulphate, progesterone, SHBG, cervical neoplasia, case–control study

## Abstract

Both parity and oral contraceptive use are associated with elevated circulating levels of sex hormones, at least transiently, and with increased risk of cervical cancer in human papillomavirus (HPV)-infected women. We directly evaluated whether elevations in the physiologic levels of these hormones predispose to the development of cervical neoplasia. We identified 67 premenopausal and 43 postmenopausal women with cervical intraepithelial neoplasia 2, 3, or cervical cancer (⩾CIN2) diagnosed during enrollment of a population-based cohort of 10 077 women. Four controls, two chosen randomly and two chosen from women testing positive for cancer-associated HPV, were matched to each case on menopausal status, age, days since last menses (pre), or years since menopause (post). Sex hormone-binding globulin, oestradiol, oestrone, oestrone-sulphate, dehydroepiandrosterone sulphate, and progesterone were measured in enrollment plasma. There was no consistent association between the sex hormones and risk of ⩾CIN2. Excluding cases with invasive disease had a minimal impact on results. Though this case–control study was based on a well-defined population, it was limited by reliance on a single measure of hormone levels taken at the time of diagnosis. Nonetheless, our results do not support the hypothesis that plasma levels of sex hormones have an important bearing on the risk of cervical neoplasia in HPV-infected women.

Research of the past two decades has established that infection with oncogenic types of human papillomavirus (HPV) is the major cause of cervical cancer (Bosch *et al*, 2002). Although infection with HPV is common, relatively few infections persist and progress to malignancy. Therefore, research has turned towards identifying factors that work together with HPV to cause cervical cancer. Among HPV-positive women, consistently identified risk factors for cervical cancer include parity and long-term oral contraceptive use (Bosch *et al*, 2002; Moreno *et al*, 2002; Munoz *et al*, 2002). While the means by which these factors are associated with an increase in risk is unclear, it could relate to elevated levels of circulating hormones such as oestrogens and progesterone. Evidence of a role for endogenous hormones in cervical carcinogenesis deriving from a small epidemiologic study (Zheng, 1990), and several laboratory studies of cervical cell lines and HPV-16 transgenic mice (Mitrani-Rosenbaum *et al*, 1989; Pater *et al*, 1994; Arbeit *et al*, 1996; Chen *et al*, 1996; Elson *et al*, 2000), stimulated us to conduct a population-based case–control study to examine the association between plasma levels of several hormones (oestradiol, oestrone, oestrone sulphate, dehydroepiandrosterone sulphate (DHEAS), progesterone), sex hormone-binding globulin (SHBG), and the risk of cervical cancer.

## MATERIALS AND METHODS

### Guanacaste cohort

**Figure 1:**
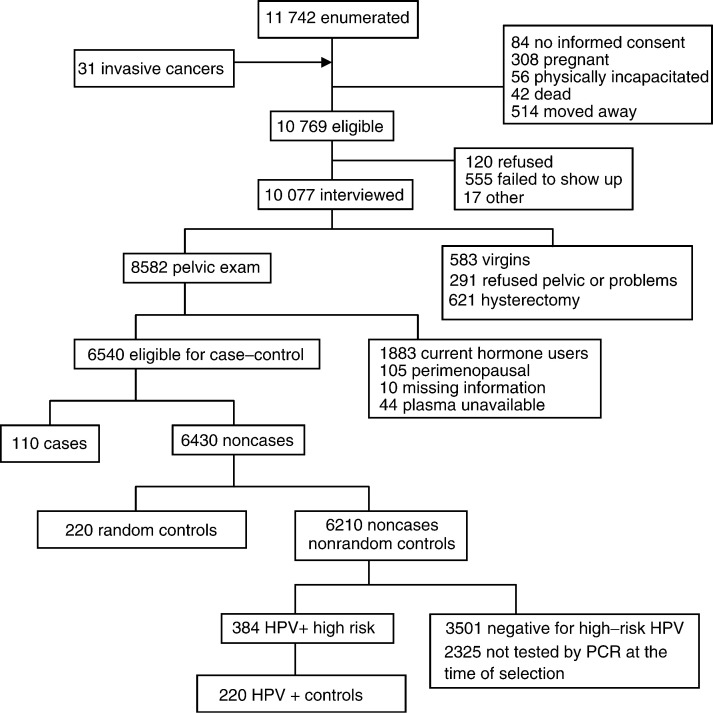
Study subject selection.

Study subjects were drawn from the enrollment phase of a population-based cohort study of cervical cancer that was launched in Guanacaste, Costa Rica, in 1993. The Guanacaste cohort has been described in detail (Herrero *et al*, 1997). The study was conducted after approval by the NCI and local institutional review boards, and all participants provided written informed consent. Censal segments were used as the primary sample unit for selection of participants, and 11 742 women over 18 years of age were enumerated (Figure 1

). In addition, since the expected number of invasive cervical cancer cases was small, all identified residents of Guanacaste newly diagnosed with invasive cervical cancer (*n*=31) during enrollment of the study in the three regional hospitals or referred to three tertiary care hospitals in San Jose were invited to participate in the study.

In all, 93.6% (*n*=10 077) of the 10 769 women eligible for interview completed a brief interview that collected information on the socioeconomic, demographic, sexual, reproductive, medical, and smoking history. A total of 8582 sexually active women with an intact uterus underwent a pelvic exam, followed by a cervical scrape with a Cervex brush that was used to prepare a conventional Pap smear. Residual cells remaining on the sampling device were rinsed in PreservCyt (Boxborough, MA, USA) and used to prepare a ThinPrep slide. A second exfoliative sample was collected in specimen transport medium (STM, Digene, Silver Spring, MD, USA) for HPV testing. Then, the cervix was painted with 5% acetic acid and two cervigrams (photographic images) were obtained.

Women with cervical abnormalities detected by visual inspection, cytology or cervicography, as well as a 2% random sample of the study population were referred for colposcopy and biopsy if indicated. The final diagnoses were based on review of cytology, cervigram, and histology. Final diagnoses included 40 women (28 identified in hospitals) with invasive cervical cancer (39 histologically confirmed, one surgically evident), 119 with histologically confirmed high-grade cervical intraepithelial neoplasia (CIN 2-3), nine with CIN 2-3 defined by reviewed and confirmed cytology, 189 with low-grade cervical intraepithelial lesions (CIN 1), 661 with equivocal lesions, and 7564 with normal final diagnoses.

In all, 99.2% of the women interviewed donated a 15 ml blood sample that was collected in a heparinised tube and kept at 1–4°C in coolers in the field. The samples were transported daily to field stations, where the plasma was isolated and frozen in aliquots at −30°C. Weekly, the samples were transported on ice packs to San Jose, stored at −70°C, and sent on dry ice to the NCI repository in Maryland, where they were stored long term at −70°C.

Cervical cells were tested for the presence of HPV using PCR with MY09/MY11 L1 consensus primers (Herrero *et al*, 2000). Laboratory personnel were blinded to the case–control status of samples. At the time HPV-positive controls were selected for the current case–control study, a total of 5703 subjects, 57.2% of the subjects with blood samples collected, had been tested for HPV using PCR. A total of 707 (12.4% of those tested) subjects tested positive for at least one high-risk type (HPV 16, 18, 31,33, 35, 39, 45, 51, 52, 56, 58, 59, 68), 704 (12.3%) tested positive for only low-risk types, and 4292 (75.3%) tested negative for HPV.

### Case–control study subjects

Subjects for this study were drawn from the 8582 women in the Guanacaste study with an interview and final diagnosis (Figure 1). Women who were perimenopausal (reported they were no longer having periods and had 1 year or less since their last menstrual period), taking exogenous hormones (93% of users were using oral contraceptives) in the month before blood was drawn, missing data, or who had fewer than two vial of plasma remaining in storage were excluded.

All 110 women diagnosed with ⩾CIN 2, who met the eligibility requirements, were chosen as cases. There were 76 cases with histologically confirmed CIN 2-3, nine cases with CIN 2–3 defined by reviewed and confirmed cytology, eight cases with invasive cervical cancer found during screening, and 17 cases with invasive cervical cancer identified in hospitals. Two sets of controls were matched to cases, each in a 2 : 1 ratio. The first set of controls, hereafter referred to as random controls, was chosen from the 6430 women with neither CIN 2–3 nor invasive cancer. The second set of controls, HPV-positive controls, was chosen from the pool of noncases and nonrandom controls, who had PCR results indicating a high-risk HPV type (*n*=384). All controls were frequency matched to cases by 5- or 10-year age group and menopausal status. Premenopausal controls were further matched to cases by days since the last menstrual period, and postmenopausal controls were further matched to cases by years since menopause.

Among premenopausal women, matching to random controls was as follows: 108 matched perfectly on days since the last menstrual period and age, and 10 matched within 3 days since the last menstrual period and 5 years of age. In all, 118 premenopausal HPV-positive controls matched to cases within 3 days since the last menstrual period. Altogether, 96 of the 118 matched within 5 years of age and 22 within 10 years of age. Eight premenopausal cases reported greater than 40 days since their last menstrual period, and were matched to 16 random and 16 HPV-positive controls with greater than 40 days since their last menstrual period. Among postmenopausal women, 73 random controls matched perfectly on years since menopause and age. The remaining 13 random controls matched within 3 years since menopause and 5 years of age. Of the 86 HPV-positive postmenopausal controls, 55 matched within 3 years since menopause and 5 years of age, 22 matched within 3 years of menopause and 10 years of age, and the remaining nine matched within 12 years since menopause and 10 years of age.

The final control groups contained the following numbers of CIN1/histologic atypia: premenopausal random *n*=6, premenopausal HPV-positive *n*=36, postmenopausal random *n*=0, postmenopausal HPV-positive *n*=6.

### Hormone assays

Plasma samples were shipped on ice to Esoterix (Endocrine Sciences, Calabasas Hills, CA, USA) for hormone analysis using in-house methods. Briefly, oestradiol, oestrone, and progesterone were measured using radioimmunoassay (RIA) following hexane : ethyl acetate extraction and column chromatography. Oestrone sulphate was measured similarly following overnight incubation with a sulphatase. Sex hormone-binding globulin was measured using a ‘sandwich’ immunoradiometric assay (IRMA), utilising a solid-phase capture antibody and a label antibody in the presence of calcium. Sex hormone-binding globulin is the major transport protein of oestradiol, and is thus inversely related to free oestradiol, the fraction of oestradiol that is not bound and thus is most biologically active. Free oestradiol was estimated by a method proposed by Sodergard *et al* (1982), using the total oestradiol measure, the SHBG measure, and assuming an albumin level of 43 g l^−1^ (Vermeulen *et al*, 1999). Dehydroepiandrosterone sulphate was measured by RIA following enzymolysis of the sulphate group. Laboratory personnel were blinded to case–control status of the samples. Repeated testing of six samples inserted blindly into each batch of samples resulted in the following overall coefficients of variation for the assays: SHBG 6.0%, oestradiol 16.9%, oestrone 15.3%, oestrone sulphate 15.5%, progesterone 17.5%, and DHEAS 8.3%. The lower limits of detection of the hormone assays were as follows: SHBG 20 nmol l^−1^, oestradiol 5 pg ml^−1^, oestrone 5 pg ml^−1^, oestrone sulphate 100 pg ml^−1^, DHEAS 10 *μ*g dl^−1^, and progesterone 100 pg ml^−1^. Postmenopausal progesterone levels are not presented.

### Statistical analysis

Data were analysed in four groups based on menopausal status and two sets of controls. The median hormone levels in cases and controls were compared using a nonparametric *k*-sample test (StataCorp, 2001). Hormone values were categorised into thirds based on the distribution in the random controls, separately for premenopausal and postmenopausal women. Relative risks were estimated by odds ratios obtained from unconditional logistic regression analysis. Relative risks obtained using conditional logistic regression were similar to those from unconditional logistic regression. Only the unconditional logistic regression results are shown. Tests for trend were obtained by assigning ordinal values to hormone tertiles and treating them as continuous variables in the model. All logistic models were adjusted for matching variables. A common model was determined for each analytic group. The common model was adjusted for factors that confounded the beta coefficient relating any single hormone and the outcome by 10% or more. Variables considered as potentially confounding factors included reproductive factors (number of pregnancies, live births, still births, miscarriages, abortions, cesarean sections, age at first pregnancy, age at menarche, oral and injectable contraceptive use, intrauterine device use, and tubal ligation), sexual behaviour, and HPV-associated variables (lifetime number of sexual partners, number of sexual partners in the last year, age at first intercourse, yeast infections, other sexually transmitted diseases, hand or foot warts), socioeconomic status variables (marital status, income, education, and number of household amenities), smoking variables, and Pap smear history.

To further control for the cyclic variation of oestrogens during the menstrual cycle in premenopausal women, we attempted to capture the midluteal rise of oestrogens by repeating analyses restricted to women thought to be in the luteal phase of their menstrual cycle based on having progesterone levels greater than 2000 pg ml^−1^ or having 17–23 days since the start of their last menstrual period.

To examine whether the results of the study were biased by inclusion of invasive cervical cancers affecting circulating hormone levels, the median hormone levels were compared in cases with invasive cervical cancer and cases with CIN 2-3. Additionally, the data were analysed with and without subjects with invasive cervical cancer.

## RESULTS

Premenopausal cases and controls were similar with respect to age, history of oral contraceptive use, lifetime number of Pap smears, and income (Table 1

**Table 1:** Characteristics of the premenopausal study population

**Characteristic**	**Cases (*N*=67)**		**Random controls (*N*=134)**		**HPV+controls (*N*=134)**	
*Age*^a^						
Mean±s.d. (range)	34.3±6.9 (19–48)		34.6±6.6 (19–49)		33.4±7.4(18–50)	

*Live births*						
0–2	18	27.7	36	27.7	47	36.7
3–4	24	36.9	54	41.5	56	43.8
5–7	18	27.7	30	23.1	16	12.5
⩾8	5	7.7	10	7.7	9	7.0

*Oral contraceptive use*						
Never	17	25.4	34	25.4	36	26.9
<2 years	20	29.9	34	25.4	47	35.1
2–4 years	14	20.9	33	24.6	24	17.9
⩾5 years	16	23.9	33	24.6	27	20.2

*Lifetime sexual partners*						
1	23	34.3	65	48.5	52	38.8
2–3	27	40.3	45	33.6	58	43.3
⩾4	17	25.4	24	17.9	24	17.9

*Lifetime Pap smears*						
0	8	11.9	7	5.2	12	9.0
1–2	14	20.9	30	22.4	34	25.4
3–5	21	31.3	51	38.1	46	34.3
⩾6	24	35.8	46	34.3	42	31.3

*Smoking*						
Never	55	82.1	117	87.3	126	94.0
Ever	12	17.9	17	12.7	8	6.0

*Tubal ligation*						
Never	47	70.2	78	58.2	94	70.2
<7 years ago	15	22.4	28	20.9	25	18.7
⩾7 years ago	5	7.5	28	20.9	15	11.2

*Income (colones/year)*						
⩽14 999	12	17.9	27	20.2	26	19.4
15 000–24 999	19	28.4	27	20.2	29	21.6
25 000–44 999	18	26.9	40	29.9	35	26.1
⩾45 000	17	25.4	35	26.1	39	29.1
Unknown	1	1.5	5	3.7	5	3.7

*HPV status*^a^						
High risk	58	86.6	16	11.9	134	100.0
Low risk	4	6.0	25	18.7	0	0
Negative	5	7.5	93	69.4	0	0

a Matching factor.

). A somewhat lower proportion of cases than random controls had undergone a tubal ligation. Cases had a greater number of lifetime sexual partners, and were much more likely to test HPV-positive than random controls. On average, the cases had more live births and were more likely to smoke than HPV-positive controls.

Postmenopausal cases and controls were of similar age, and were also similar regarding the prior use of oral contraceptives, history of tubal ligation, number of lifetime sexual partners, and years since menopause (Table 2

**Table 2:** Characteristics of the postmenopausal study population

**Characteristic**	**Cases (*N*=43)**		**Random controls (*N*=86)**		**HPV+controls (*N*=86)**	
*Age*^a^						
Mean±s.d. (range)	63.8±10.0 (45–86)		63.8±9.7 (45–86)		64.7±7.0(45–85)	

*Live births*						
0–2	1	2.3	9	11.4	11	13.3
3–4	6	14.0	6	7.6	12	14.5
5–7	15	34.9	24	30.4	16	19.3
⩾8	21	48.8	40	50.6	44	53.0

*Oral contraceptive use*						
Never	38	88.4	67	77.9	71	82.6
<2 years	2	4.7	3	3.5	5	5.8
2–4 years	1	2.3	9	10.5	7	8.1
⩾5 years	2	4.7	7	8.1	3	3.5

*Lifetime sexual partners*						
1	17	39.5	45	52.3	34	39.5
2–3	18	41.8	31	36.1	37	43.0
⩾4	8	18.6	10	11.6	15	17.4

*Lifetime Pap smears*						
0	13	30.2	25	29.1	20	23.3
1–2	17	39.5	18	20.9	37	43.0
3–5	8	18.6	25	29.1	19	22.1
⩾6	5	11.6	18	20.9	10	11.6

*Years since menopause*^a^						
Mean±s.d. (range)	13.9±1.7 (10–18)		13.6±1.6 (9–17)		14.2±1.9 (10–18)	

*Smoking*						
Never	30	69.8	71	82.6	70	81.4
Ever	13	30.2	15	17.4	16	18.6

*Tubal ligation*						
Never	40	93.0	84	97.7	84	97.7
Ever	3	7.0	2	2.3	2	2.3

*Income (colones/year)*						
⩽14 999	17	39.5	49	57.0	46	53.5
15 000–24 999	8	18.6	13	15.1	16	18.6
25 000–44 999	9	20.9	8	9.3	12	14.0
⩾45 000	1	2.3	8	9.3	4	4.7
Unknown	8	18.6	8	9.3	8	9.3

*HPV status*^a^						
High risk	34	79.1	8	9.3	86	100.0
Low risk	6	14.0	21	24.4	0	0
Negative	3	7.0	57	66.3	0	0

a Matching factor.

). The cases were slightly more likely to have smoked, and were more likely to be HPV-positive than random controls. Cases had slightly more live births, and were somewhat less likely to have had a Pap smear and more likely to have smoked than HPV-positive controls.

There were no statistically significant differences in median hormone levels between cases and controls (Tables 3

**Table 3:** Median hormone levels in premenopausal women

	**Cases**		**Ctrls**		**HPV+Ctrls**	
**Hormone**	***N***	**Median (IQR^a^)**	***N***	**Median (IQR)**	***N***	**Median (IQR)**
SHBG (nmol l^−1^)	67	80.3 (60.0)	134	70.9 (52.9)	134	75.4 (54.2)
Oestradiol (pg ml^−1^)	66	88.3 (91.5)	132	87.5 (110.7)	133	93.5 (98.0)
Oestrone (pg ml^−1^)	66	68.0 (51.0)	122	73.0 (51.5)	126	72.0 (53.0)
Oestrone sulphate (pg ml^−1^)	64	1738 (2324)	131	1776 (1536)	129	1941 (1921)
DHEAS (*μ*g dl^−1^)	67	104.1 (143.0)	134	94.5 (120.0)^b^	134	109.0 (144.5)^b^
Progesterone (pg ml^−1^)	66	1105 (6387)	134	681 (6253)	133	678 (6483)

a IQR=interquartile range.

b *P*-value=0.04 for comparison of random controls to HPV-positive controls.

and 4

**Table 4:** Median hormone levels in postmenopausal women^a^

	**Cases**		**Ctrls**		**HPV+ Ctrls**	
**Hormone**	***N***	**Median (IQR^b^)**	***N***	**Median (IQR)**	***N***	**Median (IQR)**
SHBG (nmol l^−1^)	43	76.4 (70.4)	86	81.1 (49.7)	86	69.3 (43.1)
Oestradiol (pg ml^−1^)	41	6.5 (6.0)	86	7.0 (7.0)	85	6.5 (7.0)
Oestrone (pg ml^−1^)	40	15.5 (16.5)	85	20.5 (17.0)	84	19.5 (21.0)
Oestrone sulphate (pg ml^−1^)	41	447 (346)	83	425 (280)	86	453 (271)
DHEAS (*μ*g dl^−1^)	43	37.5 (32.5)	86	45.0 (48.5)	86	42.5 (41.5)

a *P*-value>0.05 for all comparisons.

b IQR=interquartile range.

). However, progesterone levels were higher in premenopausal cases than in either random or HPV-positive controls. Among postmenopausal women, oestrone and DHEAS levels were lower in cases than in both sets of controls. Median hormone levels were similar in cases with CIN 2–3 and cases with invasive cancer (data not shown). With the exception of DHEAS levels, which were significantly higher in premenopausal HPV-positive controls than in premenopausal random controls, hormone levels were similar in both control groups among both premenopausal and postmenopausal women (Tables 3 and 4).

Tables 5

**Table 5:** Risk of ⩾CIN 2 associated with hormone levels in premenopausal women

	**Random controls**				**HPV-positive controls**			
	**Cases**	**Ctrls**	**OR^a^**	**95% CI**	**Cases**	**Ctrls**	**OR^b^**	**95% CI**
*SHBG*								
⩽55.6 nmol l^−1^	16	44	1.0		16	45	1.0	
55.7–90.1	21	45	1.1	(0.5–2.5)	21	41	1.3	(0.6–3.0)
⩾90.2	30	45	1.6	(0.7–3.4)	30	48	1.7	(0.8–3.7)
*P-trend*				*0.23*				*0.16*

*Oestradiol*								
⩽71.5 pg ml^−1^	24	44	1.0		23	47	1.0	
71.6–133.0	23	44	1.3	(0.6–2.7)	24	46	1.3	(0.6–2.8)
⩾133.1	19	44	0.7	(0.3–1.6)	19	40	0.9	(0.4–2.1)
*P-trend*				*0.39*				*0.87*

*Free oestradiol*								
⩽1.41 pg ml^−1^	28	42	1.0		28	48	1.0	
1.42–2.50	20	43	0.8	(0.4–1.7)	20	41	0.9	(0.4–1.9)
⩾2.51	17	42	0.5	(0.2–1.2)	17	34	0.8	(0.4–1.9)
*P-trend*				*0.13*				*0.63*

*Oestrone*								
⩽62.0 pg ml^−1^	27	41	1.0		27	47	1.0	
62.1–90.0	16	40	0.6	(0.3–1.3)	16	40	0.7	(0.3–1.7)
⩾90.1	19	41	0.5	(0.2–1.2)	19	39	0.9	(0.4–2.0)
*P-trend*				*0.13*				*0.77*

*Oestrone sulphate*								
⩽1254 pg ml^−1^	20	43	1.0		20	37	1.0	
1255–2287	25	44	1.3	(0.6–2.8)	25	42	1.2	(0.5–2.6)
⩾2288	19	44	0.9	(0.4–1.9)	19	50	0.7	(0.3–1.7)
*P-trend*				*0.74*				*0.45*

*DHEAS*								
⩽78.5 *μ*g dl^−1^	22	44	1.0		22	32	1.0	
78.6–114.5	18	45	0.8	(0.3–1.8)	18	39	0.7	(0.3–1.6)
⩾114.6	27	45	1.2	(0.6–2.7)	27	63	0.7	(0.3–1.5)
*P-trend*				*0.61*				*0.41*

*Progesterone*								
⩽320 pg ml^−1^	18	44	1.0		18	44	1.0	
321–2707	26	45	1.4	(0.7–3.2)	26	46	1.4	(0.6–3.1)
⩾2708	22	45	1.3	(0.6–3.1)	22	43	1.5	(0.6–3.4)
*P-trend*				*0.53*				*0.38*

a Adjusted for age (18–25, 26–30, 31–35, 36–40, 41–45, 46–50 years), days since last menstrual period (continuous), tubal ligation (never, < 7 years ago, 7+ years ago) and number of lifetime sexual partners (1, 2–3, 4+).

b Adjusted for age (18–25, 26–30, 31–35, 36–40, 41–45, 46–50 years), days since last menstrual period (continuous) and smoking (never, ever).

and 6

**Table 6:** Risk of ⩾CIN 2 associated with hormone levels in postmenopausal women

	**Random controls**				**HPV-positive controls**			
	**Cases**	**Ctrls**	**OR^a^**	**95% CI**	**Cases**	**Ctrls**	**OR^b^**	**95% CI**
*SHBG*								
⩽62.3 nmol l^−1^	19	29	1.0		19	34	1.0	
62.4–96.8	8	28	0.4	(0.1–1.3)	8	33	0.5	(0.2–1.4)
⩾96.9	16	29	0.7	(0.3–1.9)	16	19	1.5	(0.6–4.3)
*P trend*				*0.50*				*0.51*

*Oestradiol*								
⩽2.5 pg ml^−1^	11	30	1.0		11	33	1.0	
5–8.5	20	27	1.9	(0.7–5.2)	20	26	3.0	(1.1–4.3)
⩾8.6	10	29	1.0	(0.3–2.9)	10	26	1.1	(0.4–3.5)
*P-trend*				*1.0*				*0.76*

*Free oestradiol*								
⩽0.149 pg ml^−1^	13	19	1.0		13	15	1.0	
0.15–0.209	10	18	1.1	(0.3–3.7)	10	15	2.5	(0.5–11.7)
⩾0.21	7	19	0.6	(0.2–2.0)	7	22	0.3	(0.1–1.2)
*P-trend*				*0.39*				*0.09*

*Oestrone*								
⩽14.5 pg ml^−1^	18	28	1.0		18	30	1.0	
14.6–27.5	12	27	0.9	(0.3–2.5)	12	28	0.7	(0.3–2.0)
⩾27.6	10	30	0.5	(0.2–1.5)	10	26	0.6	(0.2–1.7)
*P-trend*				*0.23*				*0.34*

*Oestrone sulphate*								
⩽370 pg ml^−1^	15	27	1.0		15	28	1.0	
371–583	13	28	0.6	(0.2–1.7)	13	34	0.9	(0.3–2.6)
⩾584	13	28	0.8	(0.3–2.2)	13	24	0.9	(0.3–2.7)
*P-trend*				*0.68*				*0.90*

*DHEAS*								
⩽32 *μ*g dl^−1^	19	29	1.0		19	31	1.0	
33–61	16	28	0.9	(0.3–2.2)	16	29	0.9	(0.3–2.4)
⩾62	8	29	0.5	(0.2–1.3)	8	26	0.4	(0.1–1.2)
*P-trend*				*0.17*				*0.11*

a Adjusted for age (45–59, 60–69, 70+ years), years since menopause (continuous), number of Pap smears (0, 1–2, 3–5, 6+), smoking (never, ever), and income (<14 999, 15 000–24 999, 25 000+, unknown).

b Adjusted for age (45–59, 60–69, 70+ years), years since menopause (continuous), number of live births (0–4, 5–7, 8+), smoking (never, ever), and age at menarche (<12, 13–14, 15+).

present the risk of ⩾CIN 2 associated with hormone and SHBG levels in pre- and postmenopausal women, respectively. In premenopausal women, no statistically significant associations were seen between ⩾CIN 2 and categories of plasma levels of the various hormones and SHBG (Table 5). Increasing risk of ⩾CIN 2 in women with higher levels of SHBG was suggested, particularly in analyses using HPV-positive controls. Women with higher levels of free oestradiol or oestrone appeared to be at a somewhat decreased risk of ⩾CIN 2 relative to women with lower levels.

Among postmenopausal women, there was no clear trend of increasing or decreasing risk of ⩾CIN 2 with increasing plasma levels of hormones or SHBG (Table 6), although there was a suggestion of decreasing risk of ⩾CIN 2 in women with higher levels of oestrone or DHEAS in analyses using random and HPV-positive controls. In analyses using HPV-positive controls, women with moderate oestradiol levels were at a significantly elevated risk relative to women with low levels (OR=2.95, 95% CI 1.1–4.3), but a trend of increased risk with higher oestradiol level was not observed.

Among the 31 premenopausal cases, 61 random, and 61 HPV-positive controls, who donated blood during the luteal phase of their menstrual cycle, no significant associations between the measured hormones and risk of ⩾CIN 2 were observed (Table 7

**Table 7:** Risk of ⩾CIN 2 associated with oestrogen levels in women in the luteal phase

	**Random controls**				**HPV-positive controls**			
	**Cases**	**Ctrls**	**OR^a^**	**95% CI**	**Cases**	**Ctrls**	**OR^b^**	**95% CI**
*Oestradiol*								
⩽71.5 pg ml^−1^	6	10	1.0		6	8	1.0	
71.6–133.0	15	27	1.0	(0.3–3.8)	15	30	1.0	(0.2–4.4)
⩾133.1	10	24	0.5	(0.1–2.0)	10	22	0.8	(0.2–3.8)
*P-trend*				*0.24*				*0.72*

*Free oestradiol*								
⩽1.41	8	9	1.0		8	9	1.0	
1.42–2.50	12	25	0.5	(0.1–1.7)	12	31	0.4	(0.1–1.7)
⩾2.51	11	24	0.4	(0.1–1.4)	11	15	1.2	(0.3–5.0)
*P-trend*				*0.18*				*0.60*

*Oestrone*								
⩽62.0 pg ml^−1^	7	12	1.0		7	9	1.0	
62.1–90.0	9	19	0.7	(0.2–3.0)	9	18	0.9	(0.2–4.1)
⩾90.1	11	21	0.7	(0.2–2.8)	11	27	0.6	(0.1–2.5)
*P-trend*				*0.68*				*0.40*

*Oestrone sulphate*								
⩽1254 pg ml^−1^	6	11	1.0		6	10	1.0	
1255–2287	14	21	1.4	(0.4–5.3)	14	19	1.4	(0.3–6.1)
⩾2288	9	26	0.7	(0.2–2.6)	9	29	0.6	(0.1–2.8)
*P-trend*				*0.46*				*0.27*

a Adjusted for age (18–25, 26–30, 31–35, 36–40, 41+ years), days since last menstrual period (continuous), tubal ligation (never, <7 years ago, 7+ years ago) and number of lifetime sexual partners (1, 2–3, 4+).

b Adjusted for age (18–25, 26–30, 31–35, 36–40, 41+ years), days since last menstrual period (continuous) and smoking (never, ever).

). As in the main analysis, a decreased risk of ⩾CIN 2 in women with the highest levels of oestrogens relative to women with the lowest levels was only suggested.

Excluding subjects with invasive cervical cancer to assess the potential bias due to disease effects had no substantial effect on the findings (data not shown). Eight premenopausal cases and 32 matched controls reported more than 40 days since their last menstrual period. Exclusion of these women, mostly lactating, had no substantial effect on the results (data not shown).

## DISCUSSION

Plasma levels of SHBG, oestradiol, oestrone, oestrone sulphate, DHEAS, and progesterone were not significantly related to the risk of ⩾CIN2 in this study. To our knowledge, this is the first epidemiologic study to evaluate the role of circulating hormones and ⩾CIN2, and, to the extent that associations were observed, they conflict with our initial hypothesis that elevated levels increase the risk of cervical neoplasia.

Since multiparity and oral contraceptive use are associated both with elevated circulating hormone levels and with an increased risk of cervical cancer, we hypothesised that high physiologic oestrogen levels would adversely affect cervical carcinogenesis (Castellsague and Munoz, 2003; Smith *et al*, 2003). Results of laboratory studies suggest several different bases for such an association. In studies of cervical cells, oestradiol has been shown to upregulate the transcription of HPV E6 and E7 genes, and to stimulate the growth of HPV-positive cell lines in the absence of E6 and E7 upregulation (Mitrani-Rosenbaum *et al*, 1989; Chen *et al*, 1996). Studies of HPV-positive transgenic mice that express HPV 16 oncogenes in their basal keratinocytes have shown that chronic exposure to oestradiol is necessary for the induction of cervical tumours. Exposure to 0.05 mg oestradiol for 60 days induced tumours at the transformation zone of the mice, even though E6 and E7 levels remained low (Arbeit *et al*, 1996; Elson *et al*, 2000). A previous study in China compared the unadjusted mean hormone levels in 51 postmenopausal cervical cancer cases and 52 postmenopausal controls (Zheng, 1990). The study found higher levels of oestradiol (91.2 pg ml^−1^ in cases, 81.1 pg ml^−1^ in controls) and oestrone (116.1 pg ml^−1^ in cases, 90.2 pg ml^−1^ in controls) in cases than in controls. In contrast, our findings for total oestradiol, SHBG, oestrone, and free oestradiol fail to support the notion that high levels of oestrogens increase the risk of cervical cancer. Rather, the observed trends of increased risk in women with increasing levels of SHBG and decreased risk in women with increasing levels of oestradiol, free oestradiol, and oestrone imply that high levels of oestrogens may, if anything, be associated with a decreased risk of ⩾CIN 2.

The presence of progesterone has been hypothesised to increase the risk of ⩾CIN 2 by inducing transformation of cells by HPV-16 and upregulating HPV expression in cell lines via binding and activation of glucocorticoid response elements found on the upstream regulatory region of the virus (Pater *et al*, 1994). Use of injectible contraceptives, typically consisting of progesterone only, has also been associated with an increased risk of cervical cancer (Herrero *et al*, 1990; Hildesheim *et al*, 2001). Though premenopausal women with higher progesterone levels were at a somewhat greater risk of ⩾CIN 2 than women with lower levels, we did not observe clear trends of increasing risk with increasing progesterone level.

The ideal study of the role of hormones and cervical cancer would be prospective, with multiple measures of hormones over time and before detection of invasive disease. Such a study, however, would be extraordinarily difficult to implement in part due to the low incidence of this malignancy and because current medical practice requires intervention at earlier stages. In the light of these difficulties, a case–control study design is the reasonable first approach to explore the role of plasma hormones in cervical carcinogenesis, despite the familiar concerns about unreliable results stemming from biomarkers that may be influenced by the disease under study. We used what is arguably the optimal case group, namely confirmed precancer (CIN 2-3). The cases, identified through intensive screening of a large sample of the Guanacaste population and among Guanacaste residents with invasive cancer presenting at regional and tertiary care hospitals, comprised an unbiased sample of Guanacaste women who developed ⩾CIN 2 during enrollment. This surrogate end point poses a high cumulative risk of subsequent invasion, but is still intraepithelial and, thus, unlikely to affect the systemic hormone levels. Using subjects identified in a well-defined, large cohort allowed us to select controls that were representative of the underlying population from which most of the cases arose. DNA testing of study subjects allowed selection of a second control group of HPV-positive women, to assess whether hormones were related to the risk of ⩾CIN 2, given exposure to HPV. We were able to measure a panel of five hormones and SHBG using assays with good reproducibility.

Several issues may have contributed to our finding of no association between sex hormones and ⩾CIN 2. Even in a large population-based study such as this, the number of cases of ⩾CIN 2 available for analysis was small, particularly after separating subjects by menopausal status. Thus, we were not well-powered to detect modest risks. Despite our focus on CIN 2 and 3, the neoplasia may have influenced plasma hormone levels. However, median hormone levels were similar among cases with invasive disease and cases with CIN 2-3, results were similar after exclusion of invasive cases, and we reiterate our belief that intraepithelial lesions are unlikely to affect the circulating hormone levels. Finally, the use of a single blood sample at one point in time to characterise the long-term hormonal environment of a woman may be problematic. Endogenous hormone levels are known to vary throughout a woman's lifetime, in response to various factors such as changes in body weight, exogenous hormone use, and during menstrual cycling. We matched controls to cases by age, years since menopause (postmenopausal) and days since the last menstrual cycle (premenopausal), to account for this. Analyses including all premenopausal women and those restricted to women in the luteal phase of the cycle yielded similar results. However, we lacked information to control for other sources of variation in hormones over time, such as menstrual cycle length. Our inability to account for sources of hormone variability may have attenuated the risks and limited the ability of the study to detect some true associations (Armstrong *et al*, 1992).

In conclusion, the data fail to support hypotheses that elevated plasma levels of endogenous oestrogens or progesterone increase the risk of cervical neoplasia. If associations between endogenous hormone levels and the risk of cervical cancer truly do exist, they are likely to be modest in size. In order to reliably document such associations, future studies of squamous cervical neoplasia would require both very large numbers of subjects and a means of more accurately characterising women's long-term hormonal milieu.
